# 

**Published:** 1875-06-15

**Authors:** 


*TERMS.— Yearly Subscriptions, Three Dollars, in advance. Single Number Fifteen Cents. All Communications should be addressed to Publishers* MEDICAL EXAMINER, 65 *Randolph Street, corner State, Chicago. Postage, Fifteen Cents per annum.*

Subscribe for your Journals through our office and obtain them at

**CLUB RATES.**

| | Regular Price. | With EXAMINER. |
|---|---|---|
| The London Lancet (Am. Rep.) | $5 00 | $7 00 |
| Philadelphia Medical and Surgical Reporter, (weekly) | 5 00 | 7 00 |
| " " Times (weekly) | 5 00 | 7 00 |
| New York Medical Record (weekly) | 5 00 | 7 00 |
| " " Journal (monthly) | 4 00 | 6 00 |
| Chicago Medical Journal (monthly) | 4 00 | 6 50 |
| " Journal of Nervous and Mental Disease (quarterly) | 4 00 | 6 00 |

Any other of our home or foreign Journals furnished with THE EXAMINER at ten per cent. discount from the gross subscription price. Address:

**PUBLISHERS MEDICAL EXAMINER,**
**65 East Randolph Street, CHICAGO.**

